# Space group revsion of the triclinic polymorph of salicyl­aldehyde azine

**DOI:** 10.1107/S160053681105478X

**Published:** 2012-01-07

**Authors:** Aamer Saeed, Michael Bolte, Muhammad Arshad

**Affiliations:** a Department of Chemistry, Quaid-i-Azam University, Islamabad 45320, Pakistan; b Institut für Anorganische Chemie, J. W. Goethe-Universität Frankfurt, Max-von-Laue-Strasse 7, 60438 Frankfurt/Main, Germany; c Chemistry Division, Directorate of Science, PINSTECH, Nilore, Islamabad, Pakistan

## Abstract

The structure of the title compound, C_14_H_12_N_2_O_2_ {systematic name: 2,2′-[hydrazinediylidenebis(methanylyl­idene)]diphen­ol}, has already been determined in the triclinic space group *P* 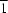 with *Z* = 4 [El-Medani, Aboaly, Abdalla & Ramadan (2004 ▶). *Spectrosc. Lett.* **37**, 619–632]. However, the correct space group should be *P*2_1_/*c* with *Z* = 4. This structure is a new polymorph of the already known monoclinic polymorph of salicyladehyde azine, which crystallizes in space group *P*2_1_/*n* with *Z* = 2. The benzene rings form a dihedral angle of 46.12 (9)°. Two intramolucular O—H⋯N hydrogen bonds occur.

## Related literature

For the structure of salicyl­aldehyde azine in *P* 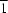 with *Z*=4, see El-Medani *et al.* (2004 ▶). For the other monoclinic polymorph of salicyladehyde azine, see for example Xue *et al.* (1994 ▶).
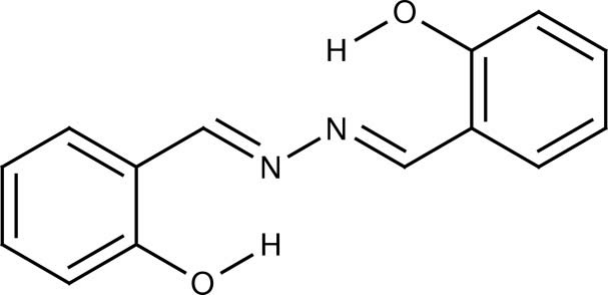


## Experimental

### 

#### Crystal data

C_14_H_12_N_2_O_2_
*M* *_r_* = 240.26
Monoclinic, 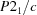

*a* = 16.3621 (11) Å
*b* = 5.9180 (4) Å
*c* = 13.1706 (9) Å
β = 113.639 (5)°
*V* = 1168.31 (14) Å^3^
*Z* = 4
Mo *K*α radiation
μ = 0.09 mm^−1^
*T* = 173 K
0.28 × 0.19 × 0.12 mm

#### Data collection

Stoe IPDS II two-circle diffractometer
14742 measured reflections
2189 independent reflections
1977 reflections with *I* > 2σ(*I*)
*R*_int_ = 0.079

#### Refinement

*R*[*F* ^2^ > 2σ(*F* ^2^)] = 0.045
*wR*(*F* ^2^) = 0.116
*S* = 1.17
2189 reflections
172 parameters
H atoms treated by a mixture of independent and constrained refinement
Δρ_max_ = 0.18 e Å^−3^
Δρ_min_ = −0.16 e Å^−3^

### 

Data collection: *X-AREA* (Stoe & Cie, 2001 ▶); cell refinement: *X-AREA*; data reduction: *X-AREA*; program(s) used to solve structure: *SHELXS97* (Sheldrick, 2008 ▶); program(s) used to refine structure: *SHELXL97* (Sheldrick, 2008 ▶); molecular graphics: *XP* (Sheldrick, 2008 ▶) and *Mercury* (Macrae *et al.*, 2006 ▶); software used to prepare material for publication: *SHELXL97*.

## Supplementary Material

Crystal structure: contains datablock(s) global, I. DOI: 10.1107/S160053681105478X/zj2044sup1.cif

Structure factors: contains datablock(s) I. DOI: 10.1107/S160053681105478X/zj2044Isup2.hkl

Supplementary material file. DOI: 10.1107/S160053681105478X/zj2044Isup3.cml

Additional supplementary materials:  crystallographic information; 3D view; checkCIF report

## Figures and Tables

**Table 1:** Hydrogen-bond geometry (Å, °)

*D*—H⋯*A*	*D*—H	H⋯*A*	*D*⋯*A*	*D*—H⋯*A*
O1—H1⋯N1	0.95 (3)	1.81 (3)	2.6454 (19)	145 (2)
O1*A*—H1*A*⋯N1*A*	0.95 (3)	1.82 (3)	2.6532 (19)	145 (2)

**Fig. 1.**
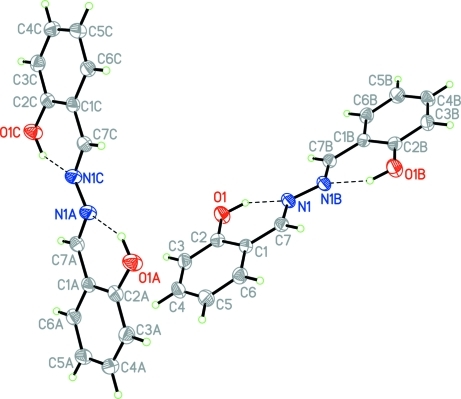
Molecular structure of title compound showing the two molecules in the asymmetric unit. Displacement ellipsoids are drawn at the 50% probability level. The atoms of the second molecule in the asymmetric unit are labelled with suffix A. Symmetry operators: (B): 2 - x,1 - y,-z, (C): 1 - x,1 - y,-z.

**Fig. 2.**
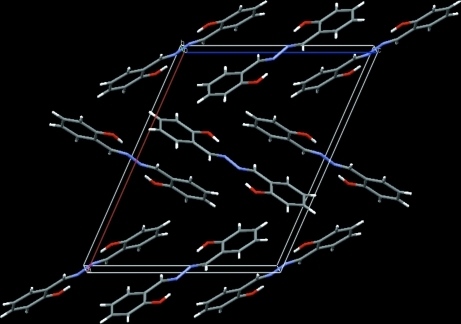
Packing diagram of the title compound with view along the b axis.

## supplementary crystallographic information

### Comment

The structure of the title compound, C_14_H_12_N_2_O_2_, has already been determined in the triclinic space group *P*1 with *Z*=4 [El-Medani, Aboaly, Abdalla & Ramadan (2004). Spectrosc. Lett. 37, 619–632]. However, the correct space group should be *P*2_1_/*c* with *Z*=4. The authors have determined the unit-cell parameters correctly, however, they thought that the structure is triclinic with four molecules in the asymmetric unit, whereas the correct description should be in the monoclinic crystal systems with two half molecules in the asymmetric unit. This structure is a new polymorph of the already known monoclinic polymorph of salicyladehyde azine, which crystallizes in the space group *P*2_1_/*n* with *Z*=2.

The title compound crystallizes with two half molecules in the asymmetric unit, both of which are located on a crystallographic centre of inversion. The molecules are essentially planar (r.m.s. deviation for all non H-atoms 0.021 and 0.018 Å, for the two molecules in the asymmetric unit). Bond lengths and angles are in the normal ranges.

In the already known monoclinic polymorph there is just one half molecule in the asymmetric unit which is located on a centre of inversion. The dihedral angle between symmetry equivalent molecules is 64.6°. The title compound, on the other hand, crystallizes with two half molecules in the asymmetric unit, which enclose a dihedral angle of 47.4°. The dihedral angle between symmetry equivalent molecules is 67.5°. Thus the difference between the two monoclinic polymorphs is the different mutual orientation of the molecules in the unit cell.

### Experimental

Hydrazine hydrate, (1 mmol) dissolved in 5 ml ethanol was added dropwise to a solution of salicylaldehyde, (2.2 mmol) in 10 ml ethanol at room temperature with continuous stirring. The reaction mixture was reflux for 4 h and completion monitored by TLC. The reaction mixture was concentrated and resulted product was separated. Single crystal of the compound, suitable for X-ray crystallography, was grown by slow evaporation from an ethyl acetate-ethanol solution (2:1). as colourless crystals: Anal. calcd. for C_14_H_12_N_2_O_2_: C, 44.95; H, C, 69.99; H, 5.03; N, 11.66 98%; found: C, 69.99; H, 5.03; N, 11.66; %.

### Refinement

The H atoms were initially located by difference Fourier synthesis. Subsequently, H atoms bonded to C atoms were refined using a riding model, with C—H = 0.95 Å and with *U*_iso_(H) = 1.2*U*_eq_(C). The H atoms bonded to O were freely refined.

### Crystal data

**Table:**

C_14_H_12_N_2_O_2_	*F*(000) = 504
*M**_r_* = 240.26	*D*_x_ = 1.366 Mg m^−^^3^
Monoclinic, *P*2_1_/*c*	Mo *K*α radiation, λ = 0.71073 Å
Hall symbol: -P 2ybc	Cell parameters from 14708 reflections
*a* = 16.3621 (11) Å	θ = 3.4–26.2°
*b* = 5.9180 (4) Å	µ = 0.09 mm^−^^1^
*c* = 13.1706 (9) Å	*T* = 173 K
β = 113.639 (5)°	Plate, light brown
*V* = 1168.31 (14) Å^3^	0.28 × 0.19 × 0.12 mm
*Z* = 4	

### Data collection

**Table:**

Stoe IPDS II two-circle diffractometer	1977 reflections with *I* > 2σ(*I*)
Radiation source: fine-focus sealed tube	*R*_int_ = 0.079
graphite	θ_max_ = 25.7°, θ_min_ = 3.4°
ω scans	*h* = −19→19
14742 measured reflections	*k* = −7→7
2189 independent reflections	*l* = −15→15

### Refinement

**Table:**

Refinement on *F*^2^	Secondary atom site location: difference Fourier map
Least-squares matrix: full	Hydrogen site location: inferred from neighbouring sites
*R*[*F*^2^ > 2σ(*F*^2^)] = 0.045	H atoms treated by a mixture of independent and constrained refinement
*wR*(*F*^2^) = 0.116	*w* = 1/[σ^2^(*F*_o_^2^) + (0.0386*P*)^2^ + 0.5421*P*] where *P* = (*F*_o_^2^ + 2*F*_c_^2^)/3
*S* = 1.17	(Δ/σ)_max_ < 0.001
2189 reflections	Δρ_max_ = 0.18 e Å^−^^3^
172 parameters	Δρ_min_ = −0.16 e Å^−^^3^
0 restraints	Extinction correction: *SHELXL97* (Sheldrick, 2008), Fc^*^=kFc[1+0.001xFc^2^λ^3^/sin(2θ)]^-1/4^
Primary atom site location: structure-invariant direct methods	Extinction coefficient: 0.029 (3)

### Special details

**Table:**

Geometry. All e.s.d.'s (except the e.s.d. in the dihedral angle between two l.s. planes) are estimated using the full covariance matrix. The cell e.s.d.'s are taken into account individually in the estimation of e.s.d.'s in distances, angles and torsion angles; correlations between e.s.d.'s in cell parameters are only used when they are defined by crystal symmetry. An approximate (isotropic) treatment of cell e.s.d.'s is used for estimating e.s.d.'s involving l.s. planes.
Refinement. Refinement of *F*^2^ against ALL reflections. The weighted *R*-factor *wR* and goodness of fit *S* are based on *F*^2^, conventional *R*-factors *R* are based on *F*, with *F* set to zero for negative *F*^2^. The threshold expression of *F*^2^ > σ(*F*^2^) is used only for calculating *R*-factors(gt) *etc*. and is not relevant to the choice of reflections for refinement. *R*-factors based on *F*^2^ are statistically about twice as large as those based on *F*, and *R*- factors based on ALL data will be even larger.

### Fractional atomic coordinates and isotropic or equivalent isotropic displacement parameters (Å^2^)

**Table:**

	*x*	*y*	*z*	*U*_iso_*/*U*_eq_	
O1	0.87613 (9)	0.1392 (2)	0.06801 (11)	0.0380 (4)	
H1	0.9110 (17)	0.204 (4)	0.0324 (19)	0.059 (7)*	
N1	0.97379 (9)	0.4503 (2)	0.02477 (11)	0.0281 (3)	
C1	0.90680 (10)	0.5083 (3)	0.15398 (13)	0.0253 (4)	
C2	0.86776 (11)	0.2920 (3)	0.14005 (13)	0.0274 (4)	
C3	0.81945 (12)	0.2302 (3)	0.20203 (14)	0.0317 (4)	
H3	0.7920	0.0857	0.1917	0.038*	
C4	0.81141 (12)	0.3789 (3)	0.27845 (14)	0.0341 (4)	
H4	0.7785	0.3352	0.3205	0.041*	
C5	0.85078 (12)	0.5913 (3)	0.29465 (15)	0.0358 (4)	
H5	0.8457	0.6917	0.3481	0.043*	
C6	0.89718 (11)	0.6543 (3)	0.23210 (14)	0.0312 (4)	
H6	0.9233	0.8005	0.2422	0.037*	
C7	0.95815 (11)	0.5834 (3)	0.09241 (13)	0.0264 (4)	
H7	0.9807	0.7334	0.1021	0.032*	
O1A	0.62345 (9)	0.8865 (2)	0.17775 (10)	0.0357 (3)	
H1A	0.5876 (18)	0.814 (5)	0.111 (2)	0.066 (8)*	
N1A	0.52522 (10)	0.5585 (2)	0.04906 (11)	0.0283 (3)	
C1A	0.59119 (10)	0.5328 (3)	0.24640 (13)	0.0248 (4)	
C2A	0.63079 (11)	0.7490 (3)	0.26375 (13)	0.0267 (4)	
C3A	0.67951 (11)	0.8257 (3)	0.37086 (14)	0.0306 (4)	
H3A	0.7069	0.9703	0.3822	0.037*	
C4A	0.68836 (12)	0.6931 (3)	0.46105 (14)	0.0331 (4)	
H4A	0.7218	0.7473	0.5339	0.040*	
C5A	0.64861 (12)	0.4804 (3)	0.44581 (14)	0.0331 (4)	
H5A	0.6539	0.3904	0.5079	0.040*	
C6A	0.60154 (11)	0.4023 (3)	0.33969 (13)	0.0284 (4)	
H6A	0.5754	0.2561	0.3294	0.034*	
C7A	0.54094 (11)	0.4405 (3)	0.13719 (13)	0.0263 (4)	
H7A	0.5191	0.2900	0.1303	0.032*	

### Atomic displacement parameters (Å^2^)

**Table:**

	*U*^11^	*U*^22^	*U*^33^	*U*^12^	*U*^13^	*U*^23^
O1	0.0503 (8)	0.0324 (7)	0.0411 (7)	−0.0115 (6)	0.0286 (6)	−0.0101 (6)
N1	0.0308 (7)	0.0300 (8)	0.0273 (7)	−0.0024 (6)	0.0155 (6)	0.0022 (6)
C1	0.0227 (8)	0.0290 (9)	0.0242 (8)	−0.0001 (6)	0.0094 (6)	0.0002 (6)
C2	0.0271 (8)	0.0298 (9)	0.0251 (8)	−0.0010 (7)	0.0103 (7)	−0.0018 (7)
C3	0.0291 (9)	0.0329 (9)	0.0348 (9)	−0.0043 (7)	0.0145 (7)	0.0030 (7)
C4	0.0287 (9)	0.0463 (11)	0.0327 (9)	0.0014 (8)	0.0180 (7)	0.0046 (8)
C5	0.0325 (9)	0.0450 (11)	0.0350 (9)	0.0001 (8)	0.0189 (8)	−0.0079 (8)
C6	0.0282 (9)	0.0319 (9)	0.0350 (9)	−0.0018 (7)	0.0141 (7)	−0.0053 (7)
C7	0.0258 (8)	0.0268 (8)	0.0263 (8)	−0.0003 (6)	0.0101 (6)	0.0019 (6)
O1A	0.0482 (8)	0.0300 (7)	0.0301 (7)	−0.0072 (6)	0.0170 (6)	0.0017 (5)
N1A	0.0332 (7)	0.0296 (8)	0.0234 (7)	−0.0005 (6)	0.0125 (6)	−0.0030 (6)
C1A	0.0224 (8)	0.0283 (8)	0.0260 (8)	0.0033 (6)	0.0121 (6)	0.0008 (6)
C2A	0.0277 (8)	0.0276 (8)	0.0282 (8)	0.0017 (7)	0.0146 (7)	0.0015 (7)
C3A	0.0286 (9)	0.0302 (9)	0.0339 (9)	−0.0025 (7)	0.0136 (7)	−0.0039 (7)
C4A	0.0289 (9)	0.0421 (10)	0.0268 (9)	−0.0001 (8)	0.0094 (7)	−0.0030 (7)
C5A	0.0325 (9)	0.0399 (10)	0.0264 (9)	0.0023 (8)	0.0114 (7)	0.0057 (7)
C6A	0.0270 (8)	0.0299 (9)	0.0293 (9)	0.0012 (7)	0.0124 (7)	0.0035 (7)
C7A	0.0273 (8)	0.0273 (8)	0.0274 (8)	0.0012 (7)	0.0142 (7)	−0.0007 (7)

### Geometric parameters (Å, °)

**Table:**

O1—C2	1.357 (2)	O1A—C2A	1.360 (2)
O1—H1	0.95 (3)	O1A—H1A	0.95 (3)
N1—C7	1.289 (2)	N1A—C7A	1.289 (2)
N1—N1^i^	1.398 (3)	N1A—N1A^ii^	1.405 (3)
C1—C6	1.400 (2)	C1A—C6A	1.403 (2)
C1—C2	1.409 (2)	C1A—C2A	1.411 (2)
C1—C7	1.452 (2)	C1A—C7A	1.447 (2)
C2—C3	1.394 (2)	C2A—C3A	1.389 (2)
C3—C4	1.382 (3)	C3A—C4A	1.382 (2)
C3—H3	0.9500	C3A—H3A	0.9500
C4—C5	1.390 (3)	C4A—C5A	1.394 (3)
C4—H4	0.9500	C4A—H4A	0.9500
C5—C6	1.377 (2)	C5A—C6A	1.377 (2)
C5—H5	0.9500	C5A—H5A	0.9500
C6—H6	0.9500	C6A—H6A	0.9500
C7—H7	0.9500	C7A—H7A	0.9500
			
C2—O1—H1	108.9 (15)	C2A—O1A—H1A	108.8 (16)
C7—N1—N1^i^	113.21 (17)	C7A—N1A—N1A^ii^	113.13 (17)
C6—C1—C2	118.53 (15)	C6A—C1A—C2A	118.12 (15)
C6—C1—C7	118.83 (15)	C6A—C1A—C7A	118.90 (15)
C2—C1—C7	122.62 (15)	C2A—C1A—C7A	122.99 (15)
O1—C2—C3	118.33 (16)	O1A—C2A—C3A	118.27 (15)
O1—C2—C1	121.93 (15)	O1A—C2A—C1A	121.73 (15)
C3—C2—C1	119.74 (15)	C3A—C2A—C1A	119.99 (15)
C4—C3—C2	120.09 (17)	C4A—C3A—C2A	120.49 (16)
C4—C3—H3	120.0	C4A—C3A—H3A	119.8
C2—C3—H3	120.0	C2A—C3A—H3A	119.8
C3—C4—C5	120.94 (16)	C3A—C4A—C5A	120.45 (16)
C3—C4—H4	119.5	C3A—C4A—H4A	119.8
C5—C4—H4	119.5	C5A—C4A—H4A	119.8
C6—C5—C4	119.08 (17)	C6A—C5A—C4A	119.22 (16)
C6—C5—H5	120.5	C6A—C5A—H5A	120.4
C4—C5—H5	120.5	C4A—C5A—H5A	120.4
C5—C6—C1	121.60 (17)	C5A—C6A—C1A	121.71 (16)
C5—C6—H6	119.2	C5A—C6A—H6A	119.1
C1—C6—H6	119.2	C1A—C6A—H6A	119.1
N1—C7—C1	121.24 (15)	N1A—C7A—C1A	121.26 (15)
N1—C7—H7	119.4	N1A—C7A—H7A	119.4
C1—C7—H7	119.4	C1A—C7A—H7A	119.4
			
C6—C1—C2—O1	−178.31 (15)	C6A—C1A—C2A—O1A	−179.65 (15)
C7—C1—C2—O1	0.3 (2)	C7A—C1A—C2A—O1A	0.5 (2)
C6—C1—C2—C3	1.2 (2)	C6A—C1A—C2A—C3A	1.0 (2)
C7—C1—C2—C3	179.82 (15)	C7A—C1A—C2A—C3A	−178.84 (15)
O1—C2—C3—C4	178.26 (15)	O1A—C2A—C3A—C4A	179.50 (16)
C1—C2—C3—C4	−1.3 (3)	C1A—C2A—C3A—C4A	−1.1 (3)
C2—C3—C4—C5	0.2 (3)	C2A—C3A—C4A—C5A	0.1 (3)
C3—C4—C5—C6	1.0 (3)	C3A—C4A—C5A—C6A	1.0 (3)
C4—C5—C6—C1	−1.0 (3)	C4A—C5A—C6A—C1A	−1.2 (3)
C2—C1—C6—C5	−0.1 (3)	C2A—C1A—C6A—C5A	0.2 (2)
C7—C1—C6—C5	−178.71 (16)	C7A—C1A—C6A—C5A	179.97 (15)
N1^i^—N1—C7—C1	−178.38 (16)	N1A^ii^—N1A—C7A—C1A	−179.39 (16)
C6—C1—C7—N1	175.52 (15)	C6A—C1A—C7A—N1A	176.00 (15)
C2—C1—C7—N1	−3.1 (2)	C2A—C1A—C7A—N1A	−4.2 (2)

Symmetry codes: (i) −*x*+2, −*y*+1, −*z*; (ii) −*x*+1, −*y*+1, −*z*.

### Hydrogen-bond geometry (Å, °)

**Table:**

*D*—H···*A*	*D*—H	H···*A*	*D*···*A*	*D*—H···*A*
O1—H1···N1	0.95 (3)	1.81 (3)	2.6454 (19)	145 (2)
O1A—H1A···N1A	0.95 (3)	1.82 (3)	2.6532 (19)	145 (2)
